# Glutathione depletion sensitizes cisplatin- and temozolomide-resistant glioma cells *in vitro* and *in vivo*

**DOI:** 10.1038/cddis.2014.465

**Published:** 2014-10-30

**Authors:** C R R Rocha, C C M Garcia, D B Vieira, A Quinet, L C de Andrade-Lima, V Munford, J E Belizário, C F M Menck

**Affiliations:** 1 Department of Microbiology, Institute of Biomedical Sciences, University of São Paulao, Av. Prof. Lineu Prestes, 1374, São Paulo 05505-900, Brazil; 2 Department of Pharmacology, Institute of Biomedical Sciences, University of São Paulo, Av. Prof. Lineu Prestes, 1374, São Paulo 05508-900, Brazil

## Abstract

Malignant glioma is a severe type of brain tumor with a poor prognosis and few options for therapy. The main chemotherapy protocol for this type of tumor is based on temozolomide (TMZ), albeit with limited success. Cisplatin is widely used to treat several types of tumor and, in association with TMZ, is also used to treat recurrent glioma. However, several mechanisms of cellular resistance to cisplatin restrict therapy efficiency. In that sense, enhanced DNA repair, high glutathione levels and functional p53 have a critical role on cisplatin resistance. In this work, we explored several mechanisms of cisplatin resistance in human glioma. We showed that cellular survival was independent of the p53 status of those cells. In addition, in a host-cell reactivation assay using cisplatin-treated plasmid, we did not detect any difference in DNA repair capacity. We demonstrated that cisplatin-treated U138MG cells suffered fewer DNA double-strand breaks and DNA platination. Interestingly, the resistant cells carried higher levels of intracellular glutathione. Thus, preincubation with the glutathione inhibitor buthionine sulfoximine (BSO) induced massive cell death, whereas *N*-acetyl cysteine, a precursor of glutathione synthesis, improved the resistance to cisplatin treatment. In addition, BSO sensitized glioma cells to TMZ alone or in combination with cisplatin. Furthermore, using an *in vivo* model the combination of BSO, cisplatin and TMZ activated the caspase 3–7 apoptotic pathway. Remarkably, the combined treatment did not lead to severe side effects, while causing a huge impact on tumor progression. In fact, we noted a remarkable threefold increase in survival rate compared with other treatment regimens. Thus, the intracellular glutathione concentration is a potential molecular marker for cisplatin resistance in glioma, and the use of glutathione inhibitors, such as BSO, in association with cisplatin and TMZ seems a promising approach for the therapy of such devastating tumors.

Malignant gliomas are the most common and aggressive type of primary brain tumor in adults. Current therapy includes surgery for tumor resection, followed by radiotherapy and/or concomitant adjuvant chemotherapy with temozolomide (TMZ) or chloroethylating nitrosoureas (CNUs). However, these protocols have limited success, and patients diagnosed with glioma have a dismal prognosis, with a median survival of 15 months and a 5-year survival rate of ~2%.^1^ Several molecular mechanisms for cell resistance to these agents have been described. Because both are alkylating agents, the repair enzyme O_6_-methylguanine-DNA methyltransferase (MGMT) is certainly a first barrier that is associated with increased tumor resistance.^2, 3^ The p53 status has also been proposed to act in an opposite manner in glioma cell resistance to TMZ or CNUs. Although p53 mutation is shown to be more resistant to TMZ treatment, owing to the induction of cell death,^4^ the p53 protein protects glioma cells after CNU treatment, most likely by improving other DNA repair systems.^5^

Cisplatin is one of the most effective anticancer drugs and is used as a first-line treatment for a wide spectrum of solid tumors, such as ovarian, lung and testicular cancer,^6^ and it is used for adjuvant therapy in gliomas.^7^ Cisplatin is a molecule formed by one platinum ion that is surrounded by four ligands at the *cis* position: two chloride atoms and two amine molecules. The mechanism of action of cisplatin is mainly based on DNA damage. Once inside the cell, cisplatin becomes activated by the substitution of one or two chloride atoms by water, a process known as aquation. Owing to this process, the drug becomes positively charged and interacts with the DNA molecule, inducing the formation of DNA adducts. Activated cisplatin preferentially binds to purine bases in the nucleophilic N7 sites, where the majority of adducts occur between two guanines on the same strand, whereas ~3–5% of cisplatin adducts react with purines at the opposite strands, forming interstrand crosslinks (ICLs). The DNA lesions, in turn, trigger a series of signal-transduction pathways, leading to cell-cycle arrest, DNA repair and apoptosis.^8^

Although relatively efficient, resistance to cisplatin, either intrinsic or acquired, during cycles of therapy is common, and overcoming tumor resistance remains the major challenge for cisplatin anticancer therapy. Cellular cisplatin resistance is a multifactorial phenomenon that may include decreased drug uptake, enhanced DNA repair capacity and higher glutathione (GSH) concentration.^9^

GSH is a highly abundant, low-molecular-weight peptide in the cell, and it is well known for its critical importance in maintaining the cellular oxidative balance as a free radical scavenger. Additionally, GSH has a protective role against xenobiotic agents once its highly reactive thiol group binds and inactivates those agents. In fact, the GSH content and glutathione *S*-transferase (GST) have long been associated with cisplatin resistance in numerous cell lines and tumor tissues.^9, 10, 11^

Considering these possible pathways, it is not clear, however, which one determinates cisplatin resistance in glioma cells. Aiming to better understand the molecular mechanisms of resistance to this drug, four human glioma cell lines with different p53 status were investigated. We showed that cellular resistance was found to be independent of p53 as well as of the DNA repair capacity of the cells. On the other hand, the GSH levels within the cell were shown to act as a decisive resistance barrier to cisplatin, reducing the induction of DNA damage in the treated cells. Also, both in an *in vitro* and *in vivo* model depletion of GSH by an inhibitor (buthionine sulfoximine, BSO) sensitized the glioma cell lines to cisplatin. Interestingly, BSO also potentiated TMZ cytotoxicity. Thus, combination with BSO, cisplatin and TMZ turned out to be an extremely powerful approach to improve cytotoxicity in glioma, thus providing an exciting alternative for glioma treatment.

## Results

### The p53 status alone is not sufficient to determine cisplatin sensitivity in glioma cells

**Figure 1:**
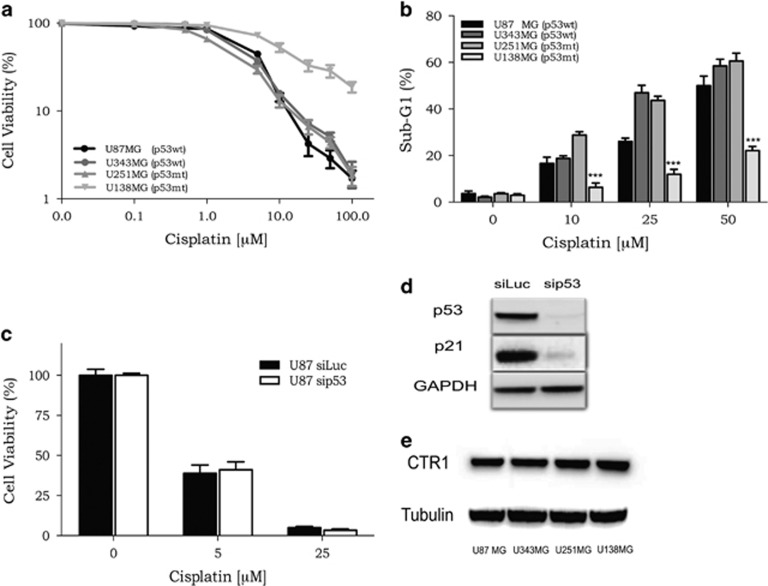
Cellular viability of glioma cells after exposure to cisplatin. (**a**) A dose–response curve of four glioma cell lines treated with increasing concentrations of cisplatin (0.01–100 *μ*M) and analyzed after 120 h treatment using the XTT assay. (**b**) A dose–response curve of the apoptotic fraction of glioma cells treated with increasing doses of cisplatin after 120 h of incubation time, analyzed as the sub-G1 population levels using flow cytometry of PI-stained nuclei. Values are mean±S.E.M. of three independent experiments, *P<0.05, **P<0.01 and ***P<0.001. (**c** and **d**) Transient transfection of siRNA targeting the *p53* gene was able to knockdown the expression of this protein, and also reduced p21 expression, although it caused no effects on the sensitivity of these cells to cisplatin treatment. (**e**) Expression of CTR1 protein was analyzed by specific antibodies in whole-cell preparations using western blot analysis. Tubulin was used as loading control. The results in E and F are representative of one out of three experiments with comparable results

It has been demonstrated that functional p53 has an opposing effect in glioma treated with TMZ or nimustine (ACNU).^4, 5^ To extend these observations to cisplatin treatment, the cell sensitivity was evaluated in glioma cell lines with different p53 status: U87MG and U343MG (p53wt) and U251MG and U138MG (p53mt). All four lineages were incubated with increasing doses of cisplatin, and, after 120 h of treatment, the cellular viability and apoptotic levels were determined. It was observed that U138MG (p53mt) cells were less sensitive to cisplatin treatment, whereas the other lineages displayed quite similar survival rates, despite their different p53 status (Figure 1a). In agreement, the analysis of the sub-G1 populations, a common methodology used for DNA fragmentation analysis because of apoptosis, after cisplatin exposure indicated lower levels for the U138MG cell line (Figure 1b). On the other hand, U251MG (p53mt) displayed a high sensitivity upon cisplatin treatment, indicated either by low cellular viability or high amounts of apoptotic cells (Figures 1a and b). In addition, when siRNA was used to silence p53 in U87MG cells, no effects were observed for cell survival after cisplatin treatment (Figures 1c and d). Those results indicate that cell sensitivity to cisplatin is independent of the p53 status in glioma cells, and other resistance factors have a more decisive role in protecting cells from cell death induced by cisplatin.

Another import resistance mechanism displayed by some tumor cells is a reduced expression level of copper transport channel (CTR1), which is involved in cisplatin uptake. Thus, lower expression of this transport channel could decrease the amount of intracellular cisplatin and could explain the resistance of the U138MG cells. However, a western blot revealed that there is no difference in the expression of CTR1 protein in the four cell lines investigated (Figure 1e).

### DNA repair is not involved in the cell resistance

**Figure 2:**
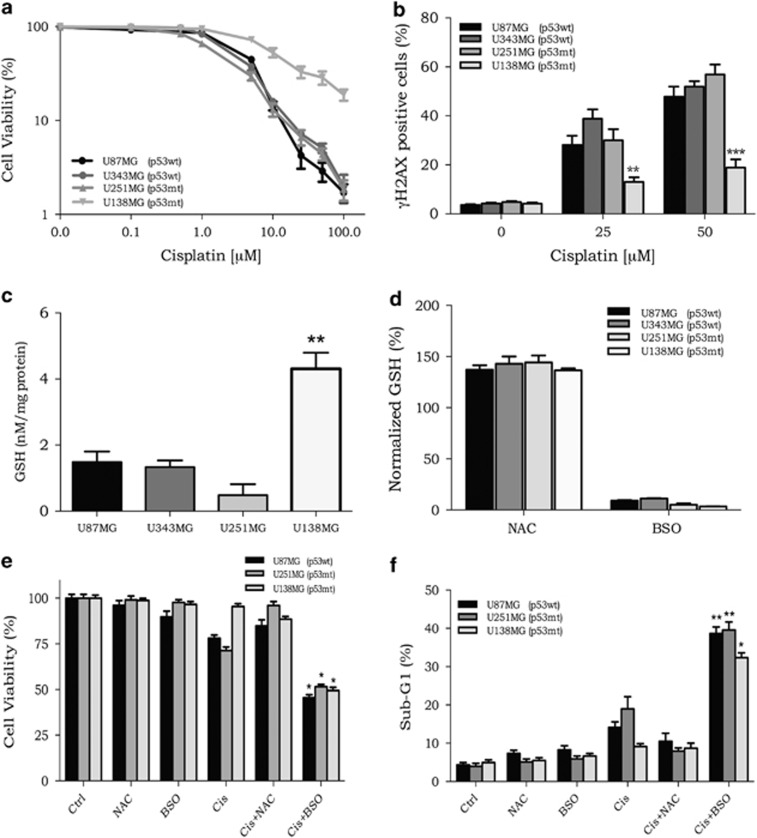
DNA repair capacity and GSH concentration in glioma cell lines. (**a**) HCR assay with a luciferase plasmid treated with increasing doses of cisplatin. (**b**) Quantification of *γ*H2AX-positive cells upon cisplatin treatment, as detected by flow cytometry. (**c**) Quantification of the basal intracellular GSH concentration in the four glioma cell lines. (**d**) Quantification of the intracellular GSH concentration after BSO or NAC incubation. (**e**) Cellular viability, as determined by the XTT assay, in cells treated with BSO or NAC combined with cisplatin. (**f**) The percentage of apoptotic cells, as measured by the sub-G1 population, after treatment with cisplatin in combination with BSO or NAC. Values are mean±S.E.M. of three independent experiments, *P<0.05, **P<0.01 and ***P<0.001

To investigate whether U138MG resistance to cisplatin was because of an enhanced DNA repair capacity, host-cell reactivation (HCR) assays were performed using a firefly luciferase gene reporter. In this assay, cisplatin-treated plasmids are transfected into the cells, and the bioluminescence that is detected, owing to reactivation of luciferase expression, is directly related to the ability of cells to remove DNA damage. The results of the HCR assay showed no difference in the bioluminescence emission from the four cell lineages, which indicates that all cell lines have a similar DNA repair capacity to remove cisplatin lesions from the damaged plasmids (Figure 2a).

During the DNA replication process, ICL lesions can lead to the formation of double-strand breaks (DSBs), these trigger the phosphorylation of histone H2AX (*γ*H2AX), which has been widely used as a DSB marker.^12, 13^ Our results pointed to a reduced percentage of *γ*H2AX-positive cells in the resistant cells (Figure 2b), indicating that fewer DSBs were generated in U138MG cells after being treated with cisplatin. Altogether, these data suggest that U138MG resistance cannot be attributed to an improved DNA repair capacity, but these cells were somehow submitted to lower amounts of DNA damage after cisplatin treatment, when compared with the other glioma cell lines.

### Depletion of GSH circumvents the resistance to cisplatin

Detoxification of cisplatin could also explain the different sensitivity of these cell lines, as this would reduce the impact of this drug. GSH may promote cisplatin detoxification, once it can bind covalently to intracellular cisplatin, preventing it from reacting with DNA, its therapeutic target. In fact, quantification of the intracellular GSH concentration of glioma cells showed that U138MG exhibited the highest levels among the investigated cell lines (Figure 2c). To modulate the cellular GSH concentration, BSO and N-acetyl-cysteine (NAC) were tested. BSO is a known inhibitor of *γ*-glutamylcysteine synthetase, an essential enzyme for the synthesis of GSH, and NAC is a GSH precursor, which increases the production of GSH within cells. As observed in Figure 2d, incubation with NAC or BSO for 18 h promoted a substantial increase or depletion, respectively, in the GSH levels of all four cell lines.

Aiming to investigate the role of GSH in cisplatin resistance, glioma cells were incubated with BSO (100 *μ*M) or NAC (25 mM) for 18 h and then treated with cisplatin. BSO or NAC alone did not result in a decrease in cell viability or in a significant increase in apoptosis (Figures 2e and f). However, it was observed that BSO in combination with cisplatin sensitized all cell lines and, more importantly, BSO pretreatment was able to overcome U138MG cisplatin resistance (Figures 2e and f). On the other hand, addition of NAC to the cell culture before cisplatin incubation was able to protect the cells from cell death triggered by cisplatin (Figure 2e). In addition, the percentage of apoptotic (sub-G1) cells was highly increased when cisplatin was combined with BSO, whereas NAC plus cisplatin protected the cells, with sub-G1 levels equivalent to those observed for the non-treated group (Figure 2f).

### BSO increases cisplatin cytotoxicity by enhancing the production of DNA damage

**Figure 3:**
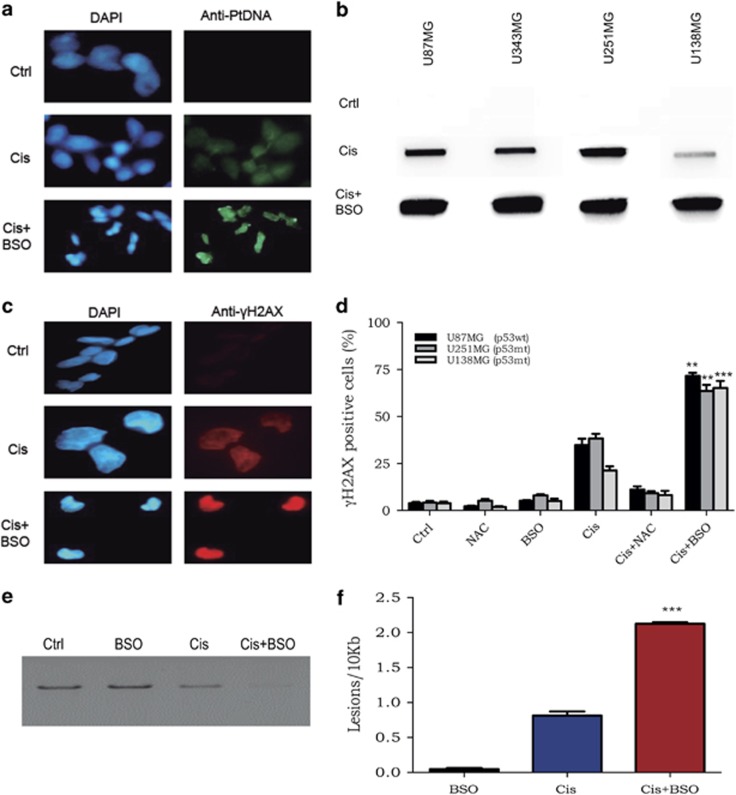
Detection of DNA damage in cisplatin-treated cells: cisplatin–DNA adducts and *γ*H2AX. (**a**) Immunofluorescence of cisplatin adducts in U138MG cells after treatment with cisplatin alone or in combination with BSO. (**b**) Immunodetection of cisplatin adducts by a slot-blot assay in U87MG and U138MG cells that were preincubated or not with BSO. (**c**) *γ*H2AX immunofluorescence of samples treated with cisplatin treatment alone or with both drugs. Those results are representative of one out of three experiments with comparable results. (**d**) Quantification of the percentage of *γ*H2AX-positive cells by flow cytometry 24 h after treatment. (**e**) Representative quantitative PCR of damage measurements of samples treated with cisplatin only or in combination with BSO. (**f**) Quantification of DNA damage in treated cells. Values are mean±S.E.M. of three independent experiments, *P<0.05, **P<0.01 and ***P<0.001

To explore the mechanisms involved in inducing cell death, the levels of DNA damage were evaluated after combined treatment with BSO and cisplatin. In GSH-depleted samples, there is an increase of cisplatin-DNA adducts as detected by immunofluorescence or slot-blot (Figures 3a and b). Furthermore, immunofluorescence staining of *γ*H2AX was higher in the samples treated with BSO plus cisplatin (Figure 3c). Flow cytometry analysis showed that BSO induced more than two times the amount of *γ*H2AX staining, which demonstrated higher levels of DNA damage after cisplatin exposure in the presence of BSO (Figure 3d). Additionally, using quantitative PCR methodology,^14^ it was observed that the cisplatin plus BSO samples generated less PCR product, which indicates a higher amount of DNA lesions (Figure 3e). In fact, estimation of the frequency of DNA lesions by Poisson distribution indicated that the combination of cisplatin and BSO induced more DNA damage than either drug alone (Figure 3f). Thus, it seems that depletion of GSH by preincubation with BSO enhances the amount of cisplatin that actually reaches the nuclear DNA, which, in turn, potentiates cisplatin-induced cytotoxicity.

### Combination of BSO, cisplatin and TMZ: a high synergistic effect *in vitro*

**Figure 4:**
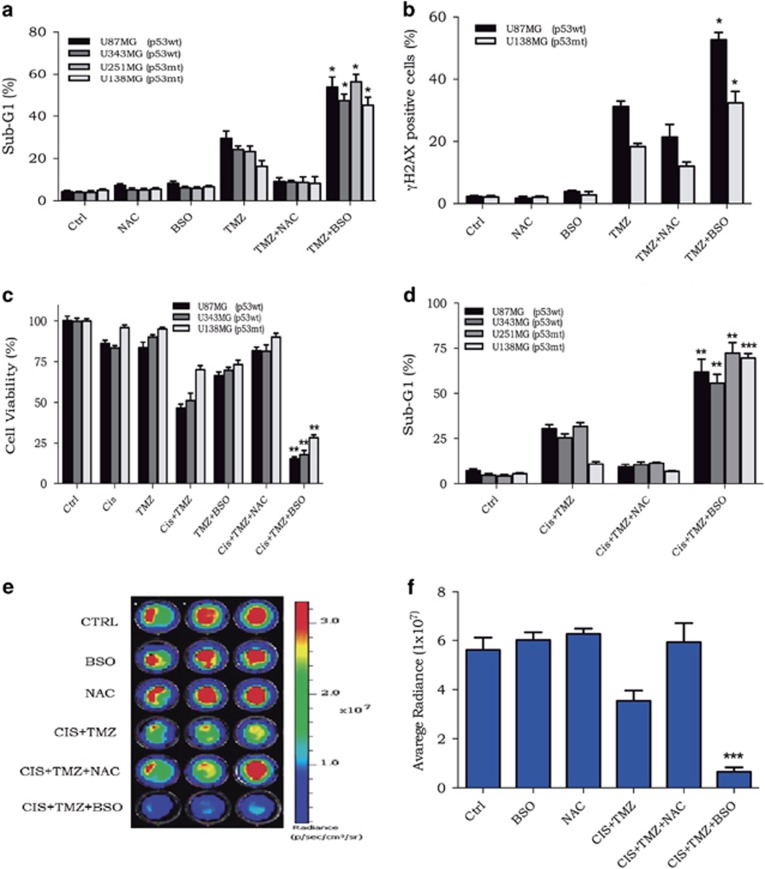
Cellular killing after exposure to BSO or NAC, cisplatin and TMZ. (**a** and **b**) Population of sub-G1 and *γ*H2AX-positive cells upon treatment with BSO or NAC followed by TMZ. (**c** and **d**) U138MG cellular viability and apoptotic population after BSO or NAC followed by cisplatin and TMZ treatment. (**e** and **f**) Bioluminescence detection and quantification of luciferase-expressing glioma (U138-Luc) cells after the indicated treatment. Values are mean±S.E.M. of three independent experiments, *P<0.05, **P<0.01 and ***P<0.001

The methylating agent TMZ is the first-line therapy drug used to treat glioma patients. As shown by previous work,^15^ BSO potentiates TMZ cytotoxicity (Figure 4a). In addition, as seen for BSO and cisplatin, the combination of BSO and TMZ greatly enhanced *γ*H2AX-positive cells (Figure 4b); in other words, GSH depletion potentiates TMZ killing effect because of the increase of DNA damage.

Based on the fact that the U138MG cell line was also resistant to TMZ treatment,^4^ the toxicity of glioma cells to BSO and cisplatin in combination with TMZ was investigated. To better verify a possible synergistic effect after drug combination, the U138MG cell line was treated with low doses of TMZ (10 *μ*M) and cisplatin (1 *μ*M) to cause minimal cellular toxicity. After 120 h of treatment with the drugs alone or in combination, cellular toxicity was determined. As it is clearly observed, the regimen treatment of BSO, cisplatin and TMZ substantially reduced cellular viability and induced high levels of apoptosis, whereas pretreatment with NAC protected cells from toxicity mediated by cisplatin plus TMZ (Figures 4c and d). Thus, a synergistic effect (*P<*0.001) was produced by combining BSO, cisplatin and TMZ in glioma cell lines.

### Combination of BSO, cisplatin and TMZ: a high synergistic effect *in vivo*

Finally, the synergistic effect of the combination of the three drugs that was observed *in vitro* was tested *in vivo*. Tumor progression was followed using *in vivo* bioluminescence imaging as this technology allows for the detection of tumor burden in a sensitive and noninvasive manner. For these experiments, glioma cell lines expressing luciferase were generated using lentivirus-derived vectors. In agreement with XTT and sub-G1 population assay data, the combination with BSO, cisplatin and TMZ was able to promote a strong increase in *in vitro* cell death, as detected by the low bioluminescence emission from the labeled cells (Figures 4e and f).

No tumor formation was detected upon inoculation of U138MG cells, and the same outcome was reported previously by others group.^16^ Therefore, U87-Luc was inoculated into the flanks of nude mice, and, after the tumor volume reached ~100 mm^3^, the animals were divided into eight groups and treated with phosphate-buffered saline (PBS), BSO, cisplatin, TMZ or combinations of these drugs. The drug treatment regimen was one dose per day for three consecutive days and then once a week for 2 weeks. During the course of treatment, no severe side effects were observed, no animals died and there was no significant weight loss because of any combinatory drug treatment (Figure 6a).

**Figure 5:**
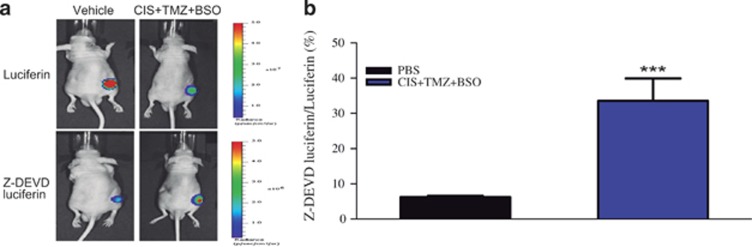
Induction of tumor cell apoptosis *in vivo* after treatment with cisplatin, TMZ and BSO. (**a**) Representative bioluminescence imaging of U87-Luc tumors after injection with Z-DEVD-aminoluciferin and luciferin. (**b**) Quantification of Z-DEVD-aminoluciferin bioluminescence signal, normalized by luciferin signal. Values are mean±S.E.M. of three independent experiments, *P<0.05, **P<0.01 and ***P<0.001

Initially, to examine directly the ability of BSO, cisplatin and TMZ combinations to induce apoptosis *in vivo*, Z-DEVD-aminoluciferin was used. This reagent is a prosubstrate that releases luciferin when it is cleaved by activated caspase-3/7. Three days after treatment, the animals were inoculated with Z-DEVD-aminoluciferin, and, 8 h later, luciferin was inoculated to measure tumor volume. Upon Z-DEVD-aminoluciferin bioluminescence normalization to the luciferin signal, the combination of BSO followed by cisplatin and TMZ significantly induced apoptosis in the nude mice bearing glioma tumors (Figures 5a and b).

**Figure 6:**
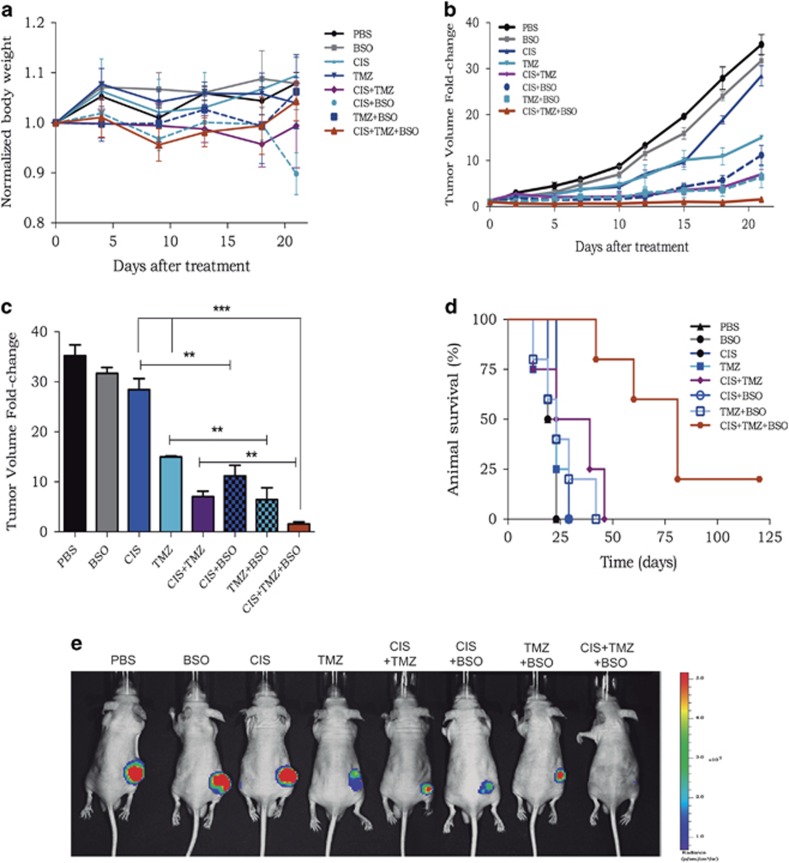
Tumor progression after treatment of animals with different chemotherapy combinations. (**a**) Time-course body weight progression was measured two times a week for 3 weeks in the eight treatment groups. (**b**) Time-course of U87-Luc tumor volume progression, as determined by caliper measurements. (**c**) Normalized tumor volume measured 21 days after the beginning of treatment. (**d**) Animal survival after treatment with different drug combinations, as indicated in the figure. Each point is the average of the tumor volume of five animals±S.E.M., *P<0.05, **P<0.01 and ***P<0.001. (**e**) Representative luciferin bioluminescence of U87-Luc tumor growth observed in animals treated with different drug combinations, as indicated, after 21 days

Remarkably, 2 weeks after the initial treatment, a strong inhibition on tumor growth progression was observed in the animal groups inoculated with BSO in combination with either cisplatin or with TMZ (Figure 6b). Moreover, this effect was much more pronounced when the animals were simultaneously inoculated with BSO, cisplatin and TMZ. In fact, at the end of treatment, the tumor volume of the control group (animals treated with PBS or BSO alone) was increased by ~35-fold in relation to that on the first day of treatment, whereas, in the group treated with the three drugs together, the average tumor volume was about the same as that at the beginning of treatment (Figures 6b, c and e). For ethical reasons, animals with large tumor burden were killed. The key result is that, when using the combination of the three drugs, there was a significant increase in animal survival by about 3-fold change and notably a full tumor regression for one of the animals (Figure 6d).

## Discussion

Nearly four decades after its FDA approval, the clinical benefits of cisplatin as an anticancer agent are unquestionable. However, tumor resistance to cisplatin still remains a huge challenge. Similarly, glioma treatment efficacy is limited and, as so, this disease remains incurable. Clearly, it is urgent and necessary to search for an alternative and to improve the existing glioma therapy protocols.

In this study, several well-known mechanisms of resistance to cisplatin were investigated in glioma cell lines, with the aim to extend the knowledge of what factors are decisive in conferring resistance to this drug and search for alternatives to achieve a more favorable outcome for glioma patients.

The tumor suppressor gene p53 is commonly correlated with cellular sensitivity to several drugs used to treat cancer. In the majority of cases, it is reported that p53 mutation leads to cellular resistance to several drugs. However, there are conflicting data concerning cisplatin treatment and p53 status in glioma cell lines.^17, 18, 19^ In our study, using four distinct cell lines, two with wild-type p53 and two with mutated p53, no correlation between p53 status and cisplatin cellular sensitivity was observed: the cell viability, as determined by XTT assays, and apoptosis (sub-G1) results revealed that U251MG cells (p53mt) are highly sensitive to cisplatin, in contrast to U138MG, which is also a p53 mutant cell line. Additionally, p53 silencing did not cause any change in cell viability upon cisplatin treatment. Our results suggest that the absence of a functional p53 alone is not sufficient to confer cellular resistance to cisplatin in glioma cells.

Cisplatin adducts induce bulky alterations in the DNA structure that disturb DNA replication, leading to cell-cycle arrest and apoptosis. Those DNA adducts are recognized and repaired by the nucleotide excision repair (NER) pathway. Thus, NER activity is a key factor to promote cellular viability after cisplatin treatment.^20^ In that sense, an enhanced DNA repair capacity to address DNA lesions caused by cisplatin is another crucial cellular mechanism of resistance. Many studies have shown that resistant cells are able to remove cisplatin adducts at an increased rate^21, 22^ or, conversely, demonstrated an association between high sensitivity and reduced DNA repair capacity.^23^ However, in the present work, no difference was observed in DNA repair capacity among the four cell lines, as indicated by the reactivation of damaged reporter gene expression. This implies that a more efficient DNA repair of cisplatin adducts cannot explain the U138MG drug resistance.

Cisplatin detoxification by GSH generally occurs by a reaction catalyzed by GST, in which GSH binds covalently to cisplatin and the formed GSH conjugates can be exported from the cells, preventing the drug from reaching its most important molecular target, DNA.^24^ Thus, DNA platination is strongly associated with cisplatin effectiveness; in other words, the higher amounts of DNA adducts, the better the treatment outcome.^10, 25^ BSO has long been used as a potent inhibitor of GSH synthesis, and previous work has shown that, in fact, BSO was able to increase cytotoxicity in several cisplatin-resistant cell lines.^9, 26^ It is also worth noting that BSO has been used in several clinical trials associated with anticancer drugs, and it is well tolerated by patients.^27, 28^ Here, inhibition of GSH synthesis by BSO was shown to overcome cisplatin resistance in glioma cells, mainly because of an increase in DNA damage induction by cisplatin.

The TMZ regimen has been shown to be effective and has rapidly replaced alkylating agents such as BCNU; therefore, it was adopted as the first-line chemotherapeutic agent for patients with newly diagnosed glioblastoma. In fact, glioma patients responded well to TMZ and, when administered in combination with radiotherapy, a significant improvement in the 2-year survival rate was observed (27% *versus* 10%).^29^ Similar to cisplatin, TMZ also targets DNA, producing several types of base lesions through methylation, and the alkylated product O^6^-methylguanine (O^6^MeG) is particularly harmful to the cells. This type of lesion is directly repaired by a suicide enzyme known as MGMT, which removes the methyl group of the DNA base and transfers it to its cysteine residue.^30^ As a consequence, the MGMT enzyme has a critical role in determining TMZ resistance; therefore, methylation of the promoter of the *MGMT* gene, which reduces its expression, is considered to be an important biomarker for drug responsiveness.^30^

In fact, Stupp *et al.*^31^ showed a strong correlation between *MGMT* promoter methylation and the effectiveness of TMZ treatment in a randomized, phase III trial. Interestingly, it has been shown that cisplatin treatment can reduce MGMT activity and expression, which is an explanation for the synergism between TMZ and cisplatin.^32^ Many clinical trials report the combination of TMZ and cisplatin as a valuable alternative for treating refractory and recurrent glioma.^33, 34, 35^

In the present work, we demonstrated that cellular resistance to TMZ was highly overcome by BSO, whereas NAC prevented TMZ cytotoxicity. This is in agreement with previous work indicating that TMZ resistance in glioma is associated with lower production of reactive oxygen species by the resistant cell line,^15^ although this does not exclude the possibility of a scavenging action of GSH also for TMZ. Recent work has also indicated that BSO can potentiate the toxicity of TMZ-induced bystander effect on glioblastoma cells^36^ and a GSH transferase inhibitor was shown to potentiate the cytotoxic effect of TMZ in melanoma cells.^37^ In this work, we further demonstrated that in GSH-depleted cells, TMZ induces a higher amount of DNA damage. Thus, as TMZ generates the DNA-methylating carbenium ions, these may be scavenged by SH-group-containing molecules, such as GSH, and this may be an important mechanism of TMZ detoxificaton within the cells.

Moreover, GSH depletion by BSO could strongly potentiate cellular death triggered by TMZ and cisplatin. Thus, the use of cisplatin and TMZ in low doses may attenuate the side effects caused by those drugs, and once combined, BSO, cisplatin and TMZ revealed to have a synergistic effect on glioma cells, which could improve the clinical outcome in patients.

Collectively, the data presented here indicated that a high intracellular GSH concentration protects against the killing effects of cisplatin. For the first time, GSH depletion associated with a drug treatment regimen with cisplatin and TMZ was demonstrated to substantially enhance cytotoxicity against glioma cells. Therefore, the intracellular GSH concentration is proposed as a potential molecular marker for cisplatin resistance in glioma, and the use of GSH inhibitors, such as BSO, in association with cisplatin and TMZ seems a promising approach for the therapy of this tumor.

## Materials and Methods

### Cell lines and culture conditions

Certified glioma cell lines U87MG and U343MG (p53 wild-type), U251MG (273Arg-His homozygous p53 mutation) and U138MG (273Arg-His heterozygous p53 mutation) were a kind gift from Bernd Kaina (Mainz, Germany), and were routinely grown in DMEM (Invitrogen, Life Technologies, Carlsbad, CA, USA) supplemented with 10% fetal calf serum (Cultilab, Campinas, Brazil) and 1% antibiotic–antimycotic at 37 °C in a humidified, 5% CO_2_ atmosphere.

### Flow cytometry for sub-G1 and *γ*H_2_AX analysis

The apoptotic response after genotoxic drug treatment was measured using the flow cytometric method of sub-G1 determination. Supernatant and attached cells were collected, washed once with PBS and fixed in 70% ethanol. Ethanol-fixed cells were stained with propidium iodide (PI) at room temperature for 1 h in PBS containing 20 *μ*g/ml PI (Sigma-Aldrich, St. Louis, MO, USA), 200 *μ*g/ml RNase A and 0.1% Triton X-100. The percentage of sub-G1 cells was calculated using the CytoSoft software (Millipore, Darmstadt, Germany). For *γ*H_2_AX immunostaining, cells were fixed with 1% formaldehyde and then with 70% ethanol. Afterwards, the cells were blocked, permeabilized and incubated with primary mouse monoclonal antibody to *γ*H2AX (Ser-139) at 1 : 1000 (Upstate Biotechnology, Upstate, NY, USA) and diluted 1 : 500 for 1 h at room temperature, followed by incubation with anti-mouse FITC secondary antibody (Sigma-Aldrich) that was diluted 1 : 200 for 1 h at room temperature.

### Immunofluorescence microscopy

Cells were plated onto coverslips and placed in a 60 mm plate. The next day, the cells were treated with 10 *μ*M cisplatin for 3 h, fixed with 4% formaldehyde, permeabilized with 0.5% Triton X-100 and blocked with 0.5% BSA and 0.3% Triton-X-100 in PBS. Thereafter, the cells were incubated with primary mouse monoclonal antibody to H2AX (Ser-139) diluted 1 : 1000 (Upstate Biotechnology) or primary antibody anti-cisplatin (CP9/19) (Abcam, Cambridge, MA, USA) that was diluted 1 : 5000 for 16 h at 4 °C. Secondary antibodies labeled with Alexa 546 (Molecular Probes, Life Technologies Carlsbad, CA, USA) or FITC (Sigma-Aldrich) were added at 1 : 2000 and incubated at room temperature for 2 h. Slides were mounted with a mounting-solution reagent containing DAPI (Vector Lab, Burlingame, CA, USA) and then analyzed using a Zeiss Axiovert 200 fluorescence microscope with AXIOVISION 4.2 software (Carl Zeiss, Jena, Germany).

### Western blot

For cellular protein detection, the following antibodies were used: mouse anti-p53 (Abcam), mouse p21 (Santa Cruz, Dallas, TX, USA), rabbit anti-SLC31A1/CTR1 (Abcam), mouse anti-tubulin (Abcam) and mouse anti-GAPDH (Santa Cruz).

### Immunoblot assay

Following extraction of genomic DNA, the samples were denatured at 100 °C for 10 min, and 500 ng per well was spotted onto nitrocellulose membranes (Bio-Rad, Hercules, CA, USA) using a slot-blot apparatus (Omniphor, San Jose, CA, USA). The membrane was baked for 2 h at 80 °C and then blocked in 5% milk that was diluted in a PBS buffer for 1 h at room temperature. Subsequently, the membranes were incubated with anti-cisplatin (CP9/19) primary antibody (Abcam) that was diluted 1 : 5000 in 5% milk-PBS overnight at 4 °C. The secondary antibody, anti-mouse IgG HRP conjugate (R&D Systems, Minneapolis, MN, USA), was diluted 1 : 2000 in 5% milk-PBS, and the membranes were incubated for 2 h at room temperature. DNA lesions were detected by adding the chemiluminescence reagent Luminata western HRP substrate (Millipore), and using ImageQuant 300 (GE Healthcare, Little Chalfont, Buckinghamshire, UK).

### HCR assay

The mammalian expression vectors pShuttle/Luc and pShuttle/RL were generated as described previously.^38^ PShuttle/Luc was treated with 10–500 nM cisplatin for 4 h at 40 °C to induce DNA damage, and 1 × 10^4^ cells were plated in 96-well dishes, in triplicate, for each point. A total of 200 ng of plasmids (180 ng pShuttle/Luc and 20 ng pShuttle/RL) was used for transfection using Lipofectamine 2000 Transfection Reagent (Invitrogen, Life Technologies). Two days after DNA transfection, luciferase activities were measured using the Dual-Glo Luciferase Assay System (Promega, Madison, WI, USA) and a Glomax-Multi+ Luminometer (Promega).

### GSH quantification

The cells were scrapped and pelleted by centrifugation at 1500 r.p.m. for 10 min. Then, the cells were incubated on ice in a 1 : 10 PBS mobile phase solution containing metaphosphoric acid 5% for 30 min. GSH content was investigated using an HPLC coupled to the electrochemical detection (ESA Thermo Scientific, Sunnyvale, CA, USA) system. The equipment was composed of 584 pumps, CoulArray detector 5600 A and UV-Vis detector SPD-10AV VP (Shimadzu, Kyoto, Japan) and was used with a Kinetix column C18 100 × 2.1 mm^2^, 2.6 *μ*m (Phenomenex, Torrance, CA, USA) maintained at a temperature of 28 °C. The mobile phase consisted of sodium phosphate 25 mM, octassulfonic acid 50 *μ*M, pH 2.6, and acetonitrile 1%. Each run was performed on an isocratic flow (0.4 ml × min^−1^) for 10 min. The data were collected at 215 nm in an UV detector, and at 400, 600 and 950 mV in an electrochemical detector.

Also, cells were seeded in opaque, 96-well plates and allowed to grow for 24 h under cell culture conditions. After 18 h of incubation with 100 *μ*M BSO or 25 mM NAC, the intracellular GSH levels were quantified using the GSH-Glo GSH Assay (Promega), as recommended by the manufacturer's instructions. Briefly, cells were washed with PBS and incubated for 30 min at room temperature in a solution containing luciferin NT substrate and GST. Then, 100 *μ*l of luciferase enzyme was added and incubated for 15 min at room temperature. Luminescence was measured using a Glomax-Multi+ Luminometer (Promega). In both methods serial dilutions of a GSH standard solution were used to generate a standard curve, and the GSH concentration was normalized to the protein concentration of each well.

### Quantitative PCR (QPCR)

The quantitative PCR (QPCR) reaction was performed as described previously.^12^ Briefly, after genomic DNA extraction and quantification by PicoGreen fluorescence (Invitrogen, Carlsbad, CA, USA), the DNA was used as template for the PCR reaction. For these, TaKaRa LA PCR (Takara Bio Inc., Mountain View, CA, USA) and the sequencing primers 5′-TGGAAACCCTGTGGGCGGATAATA-3′ and 5′-CTCCAGGCCTAAGGAGCAGCAGAA-3′ were used. The PCR products were quantified by PicoGreen fluorescence, and the cisplatin-treated (50 *μ*M) or BSO-treated samples (100 mM) were divided by control sample. The resulting ratio was the relative amplification of damaged to control samples, and the DNA lesion frequencies were estimated based on the Poisson distribution.

### Establishment of glioma expressing luciferase

The luciferase gene reporter (Luc) was cloned into the *Xho*I site of the lentiviral vector pLV-CMV-SV40-Puro (kindly provided by Prof. Dr. Silvya Engler, University of São Paulo, São Paulo, Brazil), generating pLV/Luc. This plasmid was co-transfected with three auxiliary plasmids into HEK 293 T cells using the polyethyleneimine method. The recombinant lentivirus was then used to transduce U87MG and U138MG cells, and selection was performed with incubation for 2 weeks with puromycin (1.5 *μ*g/ml), resulting in the stable-expressing luciferase glioma cell lines U87-Luc and U138-Luc.

### *In vivo* procedures

Xenograft tumors were established in 10–12-week-old, female, athymic nude mice. U87-Luc cells (3 × 10^6^) were inoculated subcutaneously in the animal's flank. Tumors were allowed to grow, and ~3 weeks after inoculation, treatment began. Tumor volume measurements were calculated according to the following formula: volume=(width^2^ × length)/2. The animals were randomized into eight treatment groups: (1) PBS; (2) BSO (450 mg/kg); (3) cisplatin (1 mg/kg); (4) TMZ (10 mg/kg); (5) TMZ+cisplatin; (6) BSO+cisplatin; (7) BSO+TMZ; and (8) BSO+cisplatin+TMZ. BSO was inoculated intraperitoneally 5 h before cisplatin or TMZ treatment for three consecutive days and then once a week for 2 weeks. All animal procedures were approved by the Ethics Animal Care and Use Committee of the Institute of Biomedical Sciences, University of Sao Paulo.

### Bioluminescence imaging

For *in vitro* luciferase assays, cells were plated in 12-well plates, allowed to attach overnight and then treated with BSO (100 *μ*M), cisplatin (1 *μ*M) and/or TMZ (10 *μ*M). After 120 h, the medium was replaced by fresh medium containing D-luciferin (150 *μ*g/ml). For *in vivo* assays, D-luciferin (150 mg/kg) was inoculated intraperitoneally into the nude mice to measure the tumor size, and Z-DEVD-aminoluciferin (50 mg/kg) was used to detect apoptosis. Bioluminescence images were obtained using the IVIS Spectrum System (Perkin-Elmer Life Sciences, Waltham, MA, USA) at the CEFAP-USP facility.

### Statistical analysis

Results represent the mean of three independent experiments, each performed in triplicate, with error bars showing the S.E.M. Statistical significance was assessed using one-way ANOVA followed by Bofferoni posttesting (GraphPad Prism 6; GraphPad Software Inc., San Diego, CA, USA) (*P<0.01, **P<0.005 and ***P<0.001).

## Abbreviations

ACNU: nimustine
BSO: buthionine sulfoximine
CNU: chloroethylating nitrosourea
CTR1: copper transport channel
DSBs: double-strand breaks
GSH: glutathione
GST: glutathione *S*-transferase
HCR: host-cell reactivation
ICL: interstrand crosslink
MGMT: O_6_-methylguanine-DNA methyltransferase
NAC: *N*-acetyl cysteine
NER: nucleotide excision repair
(O^6^MeG): O^6^-methylguanine
TMZ: temozolomide
